# HELLP Syndrome and Cerebral Venous Sinus Thrombosis Associated with Factor V Leiden Mutation during Pregnancy

**DOI:** 10.1155/2014/582890

**Published:** 2014-09-17

**Authors:** Zeynep Ozcan Dag, Yuksel Işik, Yavuz Simsek, Ozlem Banu Tulmac, Demet Demiray

**Affiliations:** Department of Gynecology and Obstetrics, Faculty of Medicine, Kirikkale University, Kirikkale, Turkey

## Abstract

Preeclampsia is a leading cause of maternal mortality and morbidity worldwide. The neurological complications of preeclampsia and eclampsia are responsible for a major proportion of the morbidity and mortality for women and their infants alike. Hormonal changes during pregnancy and the puerperium carry an increased risk of venous thromboembolism including cerebral venous sinus thrombosis (CVST). Factor 5 leiden (FVL) is a procoagulant mutation associated primarily with venous thrombosis and pregnancy complications. We report a patient with FVL mutation who presented with CVST at 24th week of pregnancy and was diagnosed as HELLP syndrome at 34th week of pregnancy.

## 1. Introduction

The pregnancy-related syndromes of preeclampsia, eclampsia, and HELLP syndrome are a group of disorders related to endothelial damage triggered by pregnancy. Preeclampsia is defined as gestational hypertension (blood pressure ±140/90 mmHg) with proteinuria >0.3 g/24 hours. It is seen in about 3–8% of all pregnancies [1]. Cerebral venous sinus thrombosis is a frequently unrecognized cause of stroke affecting predominantly young women. Its typical clinical signs include headache, visual problems, and seizures.

We report a patient with FVL mutation who presented with CVST at 24th week of pregnancy and was diagnosed as HELLP syndrome at 34th week of pregnancy.

## 2. Case Presentation

**Figure 1:**
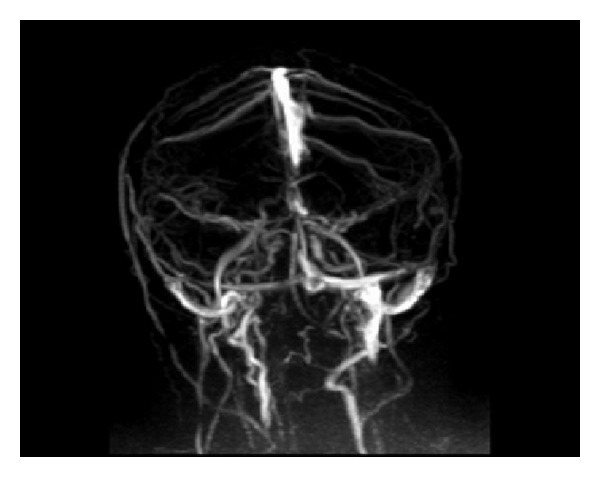
Left lateral sinus thrombosis on magnetic resonance.

A 26-year-old woman, gravida 5, abortus 3, and parity 1, was admitted to us at 24th week of gestation with headache and blurred vision. On ophthalmological examination there was bilateral papilledema and visual field loss was detected. Magnetic resonance imaging of the brain revealed CVST (Figure 1) and anticoagulant treatment (enoxaparin sodium 0.6 mL 2∗1 s.c.) was started. Hematological investigations showed thrombophilia with heterozygous factor V H1299R mutation.

At 34th week of gestation, she was hospitalized for high blood pressure, headache, nausea, and vomiting. On admission blood pressure was 220/120 mmHg and laboratory findings were as follows: aspartate transaminase (AST): 227 U/L, alanine transaminase (ALT): 176 U/L, INR: 1.3, white blood cell (WBC): 10.800 U/L, hemoglobin (Hb): 14.1 gr/dL, thrombocyte count (PLT): 153000 U/L, and 3+ proteinuria in the urine analysis. Hematological and urinary parameters were consistent with severe preeclampsia. She underwent cesarean section at 34th week of gestation due to severe preeclampsia. By cesarean section, a baby weighing 1840 g with 1 minute APGAR 9 and 5 minute 10 APGAR scores was delivered.

**Figure 2:**
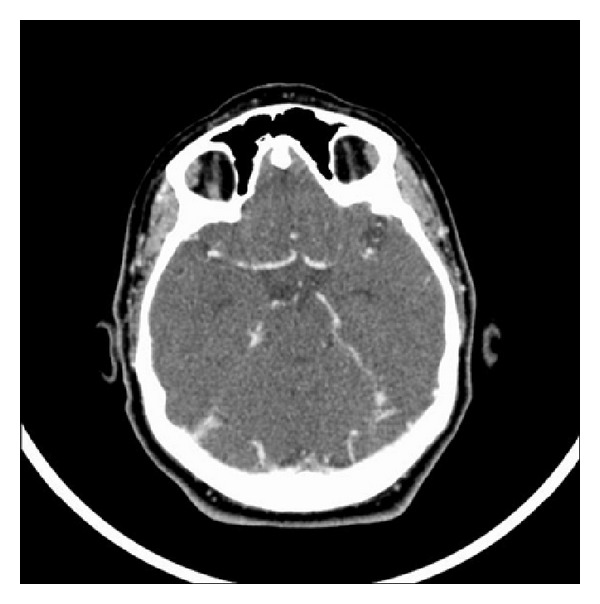
Normal CT venography.

The patient had no significant bleeding during and after cesarean section, but atony-related postpartum hemorrhage developed at 5th postoperative hour. Laboratory investigations showed WBC: 19500 U/L, Hb: 10 gr/dL, PLT: 58000 U/L, AST: 1494 U/L, and ALT: 681 U/L. Due to postpartum hemorrhage, uterine massage was performed and uterotonic drugs were administered. As bleeding continued bakri balloon was inserted. Laboratory tests were WBC: 16,600 U/L, Hb: 8.3 g/dL, PLT: 44000 U/L, AST: 1299 U/L, ALT: 489 U/L, creatinine: 1.48 mg/dL, and LDH: 2779 U/L at 7th postoperative hour. She was operated on again with total hysterectomy and bilateral hypogastric artery ligation. The patient received thirteen units of fresh frozen plasma, 3 units of platelet apheresis, 9 units of whole blood, and 3 units of packed red blood cells. At 4th postoperative day, she was discharged from the intensive care unit and monitoring continued in the service. Laboratory tests and blood pressure returned to normal at 10th postoperative day. Headache and blurred vision did not improve and bilateral papilledema still existed. The patient was started acetazolamide and enoxaparin sodium. A CT venogram showed canalised sinus thrombosis (Figure 2). She was discharged and followed up.

## 3. Discussion

Pregnancy is a hypercoagulable state. The combination of combined oral contraceptive and thrombophilia greatly increases the risk of CVST, particularly in women with hyperhomocysteinemia, FVL, and the prothrombin-gene mutation [2]. In our case, there was no history of oral contraceptive use and hyperhomocysteinemia.

The field of thrombophilia, the tendency to thrombosis, has been developed rapidly and has been linked to many aspects of pregnancy. Recurrent miscarriage has been associated with thrombophilia. It has been shown that pregnancy complications such as severe preeclampsia, intrauterine growth retardation, abruptio placentae, and stillbirth may be associated with thrombophilia [3]. Our patient had a history of recurrent abortions but had no preeclampsia and sinus thrombosis.

Thrombophilias are inherited or acquired conditions which predispose an individual to thromboembolism. Deficiencies of protein S, protein C, and antithrombin are rare and each of them is found in about 3% of patients with thrombosis. Heterozygosity for the FVL mutation is found in about 5% of the population and the mutation is responsible for 20–30% of venous thromboembolic events [4, 5]. Our patient had heterozygous factor V H1299R mutation.

The main clinical manifestations of CVST include papilledema (62%), headache (62%), hemiparesis (48%), seizures (31%), and cranial nerve palsy (7%). Patients are managed with heparin followed by warfarin during the succeeding 6 months. Death due to CVST has shown remarkable reduction because of early diagnosis and appropriate anticoagulation [6].

Patients with CVST may develop—as well as sometimes present with—chronic intracranial hypertension with headache and papilledema. The priority is prevention of visual function loss; intracranial hypertension should be controlled with acetazolamide and occasionally with repeated lumbar punctures if vision is still threatened. Refractory cases may need a cerebrospinal fluid shunting procedure [7]. Our patient did not improve with medical treatment and we are planning a shunt procedure.

Thrombophilia and CVST are rarely encountered conditions during pregnancy and augment the risk of life-threatening maternal complications and adverse perinatal outcomes in preeclamptic patients [8]. Therefore, the etiology of thrombophilia should be investigated in a timely manner. Further signs of increased intracranial pressure should be monitored closely.
